# Pharmacokinetics-Based Chronoefficacy of *Semen Strychni* and Tripterygium Glycoside Tablet Against Rheumatoid Arthritis

**DOI:** 10.3389/fphar.2021.673263

**Published:** 2021-05-24

**Authors:** Jingpan Lin, Lu Gao, Yanke Lin, Shuai Wang, Zemin Yang, Shujing Ren, Min Chen, Baojian Wu

**Affiliations:** ^1^ College of Pharmacy, Jinan University, Guangzhou, China; ^2^ Institute of Molecular Rhythm and Metabolism, Guangzhou University of Chinese Medicine, Guangzhou, China

**Keywords:** rheumatoid arthritis, semen strychni, tripterygium glycoside tablet, chronoefficacy, dosing time, pharmacokinetics

## Abstract

Rheumatoid arthritis is a systemic autoimmune disease characterized by synovial inflammation and bone destruction. Identifying drugs with time-varying efficacy and toxicity, and elucidating the mechanisms would help to improve treatment efficacy and reduce adverse effects. Here, we aimed to determine the chronoefficacy of *semen strychni* (SS) and tripterygium glycoside tablet (TGT) against rheumatoid arthritis in mice, and to investigate a potential role of circadian pharmacokinetics in generating chronoefficacy. SS extract and TGT suspension were prepared with ultrasonication. Effects of SS and TGT on collagen-induced arthritis (CIA) were evaluated by measuring TNF-α and IL-6 levels. SS dosed at ZT18 was more effective in protecting against CIA than drug dosed at ZT6 (i.e., lower levels of key inflammatory factors at ZT18 than at ZT6). This was accompanied by higher systemic exposure levels of strychnine and brucine (two main putative active ingredients of SS) in ZT18-treated than in ZT6-treated CIA mice. TGT dosing at ZT2 showed a better efficacy against CIA as compared to herb doing at ZT14. Consistently, ZT2 dosing generated a higher exposure of triptolide (a main putative active ingredient of TGT) as compared to ZT14 dosing in CIA mice. Moreover, strychnine, brucine, and triptolide significantly inhibited the proliferation of fibroblast-like synoviocytes, and reduced the production of TNF-α and IL-6 and the mRNAs of TNF-α, IL-6, COX-2, and iNOS, suggesting that they possessed an anti-arthritis activity. In conclusion, SS and TGT display chronoefficacy against rheumatoid arthritis in mice, that is attributed to circadian pharmacokinetics of main active ingredients. Our findings have implications for improving treatment outcomes of SS and TGT *via* timed delivery.

## Introduction

Rheumatoid arthritis is a systemic autoimmune disease characterized mainly by synovial inflammation and bone destruction, and is one of the main causes of human labor loss and disability (Majithia and Geraci, 2007). Although the pathogenesis of rheumatoid arthritis is poorly understood, inflammatory factors including tumor necrosis factor (TNF)-α, interleukin (IL)-1β, and IL-6 as well as excessive proliferation of synovial cells [fibroblast-like synoviocytes (FLS)] are responsible for bone and cartilage erosions, playing a crucial role in development and progression of this disease (Aupperle et al., 1998; Roccaro et al., 2005). Moreover, angiogenesis is important for leukocyte extravasation and thus for pathogenesis of RA (Szekanecz et al., 1998). Therapeutic agents for rheumatoid arthritis include non-steroidal anti-inflammatory drugs (e.g., aspirin, ibuprofen, and celecoxib) (Alaaeldin et al., 2021), slow-acting anti-rheumatism drugs (e.g., penicillamine and hydroxychloroquine) (Munro and Sturrock., 1995), and adrenocorticosteroids (e.g., cortisone, hydrocortisone and prednsion) (Boutet et al., 2021). However, most of them display long-term adverse effects and toxicity (Munro and Sturrock., 1995; Alaaeldin et al., 2021; Boutet et al., 2021). Therefore, new strategies are needed to improve the treatment outcomes of anti-arthritis drugs and to reduce toxicity.

*Semen strychni* (SS) is the dry ripe seed of Strychnos nux-vomica L (Loganiaceae) and Strychnos Pierriana A.W.Hill which are mainly distributed in China and south Asia (Chen et al., 2011). It is widely used to treat various joint pain and inflammation in Asian countries (Pharmacopoeia of the People’s Republic of China, 2005). In western countries, the extract of semen strychni is often times used as a homeopathic medicine (Jeffrey, 1987). Alkaloids are the main active and toxic constituents of SS (Guo et al., 2018). To date, 47 alkaloids have been identified from SS, among which strychnine and brucine account for 50–70% of total alkaloid content (Guo et al., 2018). Many studies have shown that strychnos alkaloids, particularly, strychnine and brucine, possess a significant anti-inflammatory property (Yin et al., 2003; Saraswati and Agarwal, 2013; Wu et al., 2017). For example, strychnine inhibits inflammatory angiogenesis in mice *via* downregulation of vascular endothelial growth factor (VEGF), TNF-α, and TGF-β (Saraswati and Agarwal, 2013). Brucine reduces the expression of TNF-α and VEGF to inhibit the abnormal proliferation of FLS, thereby alleviating the symptoms related to rheumatoid arthritis (Yin et al., 2003; Saraswati and Agarwal, 2013).

Tripterygium glycoside tablet (TGT) is a traditional Chinese medicine, isolated from the plant Tripterygium wilfordii Hook F (TwHF) that is widely used in the treatment of autoimmune and inflammatory diseases such as systemic lupus erythematosus and rheumatoid arthritis (Ma et al., 2007). The anti-arthritis effects of TwHF have been recognized for a long time in China. TwHF significantly inhibits inflammatory molecules including IL-6, TNF-α, and COX-2 which are up-regulated in the development and progression of rheumatoid arthritis (Li et al., 2015). In fact, TwHF has an advantage in the treatment of rheumatoid arthritis compared to other drugs such as corticosteroids due to a better therapeutic effect and low toxicity (Li et al., 1996). Triptolide, tripdiolide, and triptonide are the most abundant components, and also the main bioactive ingredients of TwHF (Bao and Dai, 2011). Of note, triptolide inhibits the activity of NF-kB in FLS, thereby inhibiting the expression of COX-2, iNOS and PGE-2 and reducing the production of NO (Bao and Dai, 2011). Also, it inhibits the secretion of inflammatory cytokines such as TNF-α, IL-1β, and IL-6 to attenuate inflammatory responses (Matta et al., 2009).

Almost all organisms on Earth have developed circadian clock system to adapt to diurnal changes in the environment (e.g., sunlight, temperature, and humidity). Due to the actions of the clock system, most facets of physiology and behaviors display circadian rhythms, for instance, the sleep-wake cycle, cardiovascular activity and body temperature (Ohdo, 2003). At the molecular level, circadian clock consists of a series of clock genes that are well conserved across species (Harmer et al., 2001). In mammals, the core clock genes are BMAL1 (brain and muscle Arnt-like protein-1) and CLOCK (circadian locomotor output cycles kaput), which activate the expression of PERs (periods) and CRYs (cryptochromes). Once reaching a high level, PERs and CYRs in turn inhibit the transcriptional activities of BMAL1 and CLOCK, constituting a negative feedback mechanism (Dibner et al., 2010). The nuclear receptors REV-ERBs repress, whereas RORs activate, the transcription of BMAL1, thereby completing the core clock system (Wang et al., 2020). Intriguingly, circadian clock has been shown to regulate drug pharmacokinetics, resulting in time-varying drug efficacy and/or toxicity (Ohdo, 2003; Lin et al., 2019a; Yang et al., 2020). Circadian pharmacokinetics is often times associated with diurnal expression of drug-metabolizing enzymes (Gachon and Firsou, 2011; Zhao et al., 2020a). For example, diurnal CYP3A11 regulated by BMAL1 accounts for circadian metabolism and chronotoxicity of many drug substrates such as aconitine, triptolide, and brucine (Lin et al., 2019a; Yang et al., 2020).

Clinical trials have demonstrated that chronotherapy (or time-based therapy, an emerging concept in the field of therapeutics) contributes to improved treatment outcomes of existing drugs (Khan et al., 2009; Khalifeh, 2017). Therefore, uncovering dosing time-dependent drug efficacy (chronoefficacy) and/or toxicity (chronotoxicity) and understanding the underlying mechanisms can help to improve drug efficacy and to reduce drug toxicity. In this study, we aimed to assess dosing time-dependent efficacy of SS and TGT against collagen-induced arthritis (CIA, a model of rheumatoid arthritis) in mice, and to investigate the role of pharmacokinetics in generating chronoefficacy. We found that the efficacy of SS and TGT depended on the dosing time in CIA mice. SS and TGT chronoefficacy were associated with circadian pharmacokinetics of main active ingredients (i.e., brucine and strychnine for SS and triptolide for TGT).

## Materials and Methods

### Materials

*Semen strychni* (SS) was purchased from TongRenTang Pharmaceutical Group (Beijing, China) and validated by Prof. Baojian Wu (Guangzhou University of Chinese Medicine, Guangzhou, China). A voucher specimen (No. 202009-5) for *semen strychni* was deposited at College of Pharmacy, Jinan University. Tripterygium glycoside tablet (TGT, Z42021212) was purchased from Huangshi Feiyun Pharmaceutical (Hubei, China). Strychnine (purity: 98.52%, MUST-18120411) and brucine (purity: 99.43%, MUST-19090508) were purchased from Must Biotechnology (Chengdu, China). Triptolide (purity: ≥98%, 38748-32-2) and celastrol (purity: ≥98%, 34157-83-0) were purchased from Aladdin Chemicals (Shanghai, China). Biochemical assay kits for creatinine, alanine aminotransferase (ALT) and aspartate aminotransferase (AST) were purchased from Jiancheng Bioengineering Institute (Nanjing, China). CK-BB ELISA kit was obtained from Mlbio Bio-technology (Shanghai, China). RNAiso Plus reagent was purchased from Takara (Shiga, Japan). ChamQ Universal SYBR qPCR Master Mix was purchased from Vazyme (Nanjing, China). ELISA kits for TNF-α and IL-6 were obtained from Neobioscience Technology (Shenzhen, China). Freund’s complete adjuvant (CFA) and Freund’s incomplete adjuvant (IFA) were purchased form Yuanye Bio-technology (Shanghai, China).

### Preparation of SS Extract and TGT Suspension

Raw SS (10 g, the dry ripe seed of *Strychnos nux-vomica* L.) was ground into fine powder (particle size: 100–200 mesh), and suspended in 50% ethanol (100 ml). This was followed by ultrasonication for three times at room temperature, 1 h each time. After each ultrasound, the filtrate was filtered by filter paper (pore size: 30–50 μm). The combined filtrate (three times, 300 ml) was dried in vacuum at 50°C (Gao et al., 2021). Concentrated extract (15 ml) was stored at 4°C prior to use. TGT was ground to fine powder (particle size: 100–150 mesh) and suspended in 0.5% CMC-Na solution. This was followed by ultrasonication for about 30 min until a uniform dispersing solution was formed. The uniform dispersing solution was stored at 4°C prior to use (Zhao et al., 2020b).

### Animals

C57BL/6 male mice were obtained from HFK Bio-technology (Beijing, China) and maintained under a 12 h light/12 h dark cycle [light on at 7 AM (= ZT0), light off at 7 PM (= ZT12)] with free access to food and water. Mice aged 8–12 weeks were used for experiments. All experiments were performed using protocols approved by the Institutional Animal Care and Use Committees of Guangzhou University of Chinese Medicine (Guangzhou, China).

### Collagen-Induced Arthritis

Arthritis was induced in mice by immunization with type II collagen dissolved in 0.1 M acetic acid (2 mg/ml) and emulsified with an equal volume of Freund’s adjuvant as previously described (Inglis et al., 2008). On day 0, each mouse was intradermally immunized with Freund’s complete adjuvant (CFA) emulsified collagen II at the tail end and back. On day 21, mice were boosted with Freund’s incomplete adjuvant (IFA) emulsified collagen II. After the second immunization, mice showing macroscopic signs of arthritis (with a success rate of ∼65%) were selected for further study.

### Isolation, Culture and Treatment of Murine FLS

FLS cells were isolated from the foot joints of CIA mice as previously described (Armaka et al., 2009). Cells were cultured in Dulbecco’s Modified Eagle Medium supplemented with 10% fetal bovine serum at 37°C in a humidified 5% CO_2_ atmosphere. Two sets of assays were performed with FLS. In the first set (cytotoxicity) of assays, cell viability was determined by using cell counting kit-8 (CCK-8) according to the manufacturer’s protocol (Cat. No. GK10001, GLPBIO). In brief, cells were seeded into a 96-well plate. 24 h later, cells were treated with brucine (0, 1.56, 3.13, 6.25, 12.5, 25, 50, 100, 200, and 400 μg/ml) or strychnine (0, 1.56, 3.13, 6.25, 12.5, 25, 50, 100, 200, and 400 μg/ml) or triptolide (0, 1.56, 3.13, 6.25, 12.5, 25, 50, 100, 200, and 400 ng/ml) or vehicle. After another 24 h, cells were collected for viability determination (He et al., 2019; Tang et al., 2019). In the second set of assays, cells were seeded into a 6-well plate. 24 h later, cells were treated with brucine (50 μg/ml) or strychnine (50 μg/ml) or triptolide (6.25 ng/ml) or vehicle. After 24 h, cells were collected for qPCR and determination of inflammatory factors.

### Assessment of Drug Effects

Therapeutic effects of SS and TGT were assessed using CIA mice. For dose-response experiments, CIA mice were randomly divided into seven groups (*n* = 5 mice in each group). These groups of mice were gavaged with SS extract (20, 40, and 80 mg/kg) or TGT suspension (15, 30, and 45 mg/kg) or vehicle at ZT2 once daily for 7 days. On day 8, mice were sacrificed at ZT2 to collect plasma and hind foot samples (Chen et al., 2011). For chronoefficacy experiments, CIA mice were gavaged with SS extract (40 mg/kg) or TGT suspension (30 mg/kg) or vehicle (control group) at each circadian time point (ZT2, ZT6, ZT10, ZT14, ZT18 or ZT22) once daily for 7 days. On day 8, mice were sacrificed at ZT2 to collect plasma and hind foot samples.

### Histopathology

Joint samples were fixed with paraformaldehyde, decalcified with sodium citrate, embedded with paraffin and sectioned at 6 mm, followed by hematoxylin and eosin (H&E) staining. Synovial hyperplasia, inflammatory cell infiltration, and cartilage damage were assessed as previously described (Feuerherm et al., 2019). Disease severity was graded from 0 to 4 using a scoring criterion (0, normal; 1, mild: 2, moderate; 3, marked; 4, severe). In the histological images, black arrow indicated articular periosteal hyperplasia, with a large number of infiltrating inflammatory cells (lymphocytes mainly); red arrows indicated synovial connective tissue hyperplasia, with a large number of infiltrating inflammatory cells (lymphocytes mainly); yellow arrow indicated local erosion of articular cartilage; green arrow indicated trabecular bone surrounded with a large number of osteoblasts.

### Pharmacokinetic Studies

Pharmacokinetic experiments were performed with CIA mice. Mice were randomly divided into two groups (SS and TGT groups). Mice in the SS group were gavaged with SS extract (40 mg/kg) at ZT6 or ZT18. Plasma samples were then collected at predetermined time points (i.e., 5, 7.5, 15, 30, 60, 360, and 720 min) by means of orbital plasma collection. Mice in the TGT group were gavaged with TGT suspension (30 mg/kg) at ZT2 or ZT14. Plasma samples were collected through retro-orbital bleeding at predetermined time points (i.e., 5, 15, 30, 60, 120, 360, and 720 min). All plasma samples were processed for UPLC-QTOF/MS analysis as previously described (Zhao et al., 2020a; Gao et al., 2021). AUC (area under the curve) and other pharmacokinetic parameters were obtained by using WinNonlin software (Pharsight, Cary, NC, United States) with the non-compartmental method.

### Biochemical Analysis

TNF-α and IL-6 in biological samples were measured by using ELISA kits (EMC102a and EMC004.96) according to the manufacturer’s protocol (Neobioscience, Shenzhen, China). CK-BB in biological samples was measured by using ELISA kit (ml026271) according to the manufacturer’s protocol (Mlbio, Shanghai, China). Creatinine, ALT and AST were determined by using for their assay kits (C011-2-1, C009-2-1, and C010-2-1) according to the manufacturer’s protocol (Jiancheng Bioengineering, Nanjing, China).

### qPCR Assay

RNA was extracted from biological samples using RNAiso Plus reagent and reverse-transcribed into cDNA by PrimeScript RT Master Mix (Vazyme, Nanjing, China). SYBR Green PCR Master Mix (Vazyme, Nanjing, China) was then used for amplification reactions (an initial denaturation at 95°C for 5 min, 40 cycles of denaturation at 95°C for 15 s, annealing at 60°C for 30 s, and extension at 72°C for 30 s). *Hmbs* was used as an internal control. The 2^−ΔΔCT^ method was used to calculate relative gene expression (Lin et al., 2019a; Yu et al., 2019). The primers are listed in Table 1.

**TABLE 1:** Primer sequences for quantitative real-time PCR (qPCR).

Gene	Forward (5′-3′ sequence)	Reverse (5′-3′ sequence)
*IL-6*	ATC​CAG​TTG​CCT​TCT​TGG​GAC​TGA	TAA​GCC​TCC​GAC​TTG​TGA​AGT​GGT
*TNF-*α	AGG​GTC​TGG​GCC​ATA​GAA​CT	CCA​CCA​CGC​TCT​TCT​GTC​TAC
*COX-2*	AAC​CCA​GGG​GAT​CGA​GTG​T	CGC​AGC​TCA​GTG​TTT​GGG​AT
*INOS*	GCT​CAA​GGA​GTA​TCG​GGT​CAT	GTA​GGG​CTC​ATT​CAC​CAG​GA
*Hmbs*	CCG​AGC​CAA​GGA​CCA​GGA​TA	CTCCTTCCAGGTGCCTCAGA

### UPLC-QTOF/MS Analysis

Strychnine, brucine, triptolide and their metabolites were quantified using a UPLC-QTOF/MS system (Waters, Milford, MA, United States) consisting of a Waters ACQUITY UPLC and a Xevo G2 QTOF/MS as described in our previous publications (Zhao et al., 2020b; Gao et al., 2021). Chromatographic separation was performed on an ACE UltraCore SuperC18 column (Phenomenex, Torrance, CA, United States) with a flow rate of 0.3 ml/min. The mobile phases were 0.1% formic acid (mobile phase A) and 0.1% formic acid in acetonitrile (mobile phase B). The gradient elution program was 5% B at 0–0.5 min; 5–90% B at 0.5–4 min; 90% B at 4–4.5 min; and 90–5% B at 4.5–5 min. Mass spectrometer was operated in positive ESI mode. Strychnine, brucine, pseudostrychnine, pseudobrucine, dihydroxystrychnine, triptolide, and celastrol were respectively quantified by using extracted ion chromatograms of *m/z* 335.17, 395.19, 350.16, 410.18, 367.16, 361.16, and 451.28 Da, with a mass window of ±0.05 Da (Supplementary Table S1). The representative extracted ion chromatograms are provided in Supplementary Figures S1, S2.

### Statistical Analysis

Data are presented as mean ± SD (standard deviation). Student’s *t* test was used to test for mean differences between two groups. One-way or two-way ANOVA followed by Bonferroni *post hoc* test was used for multiple group comparisons. The level of significance was set at *p* < 0.05 (*).

## Results

### Therapeutic Effects of SS and TGT on Collagen-Induced Arthritis

**FIGURE 1:**
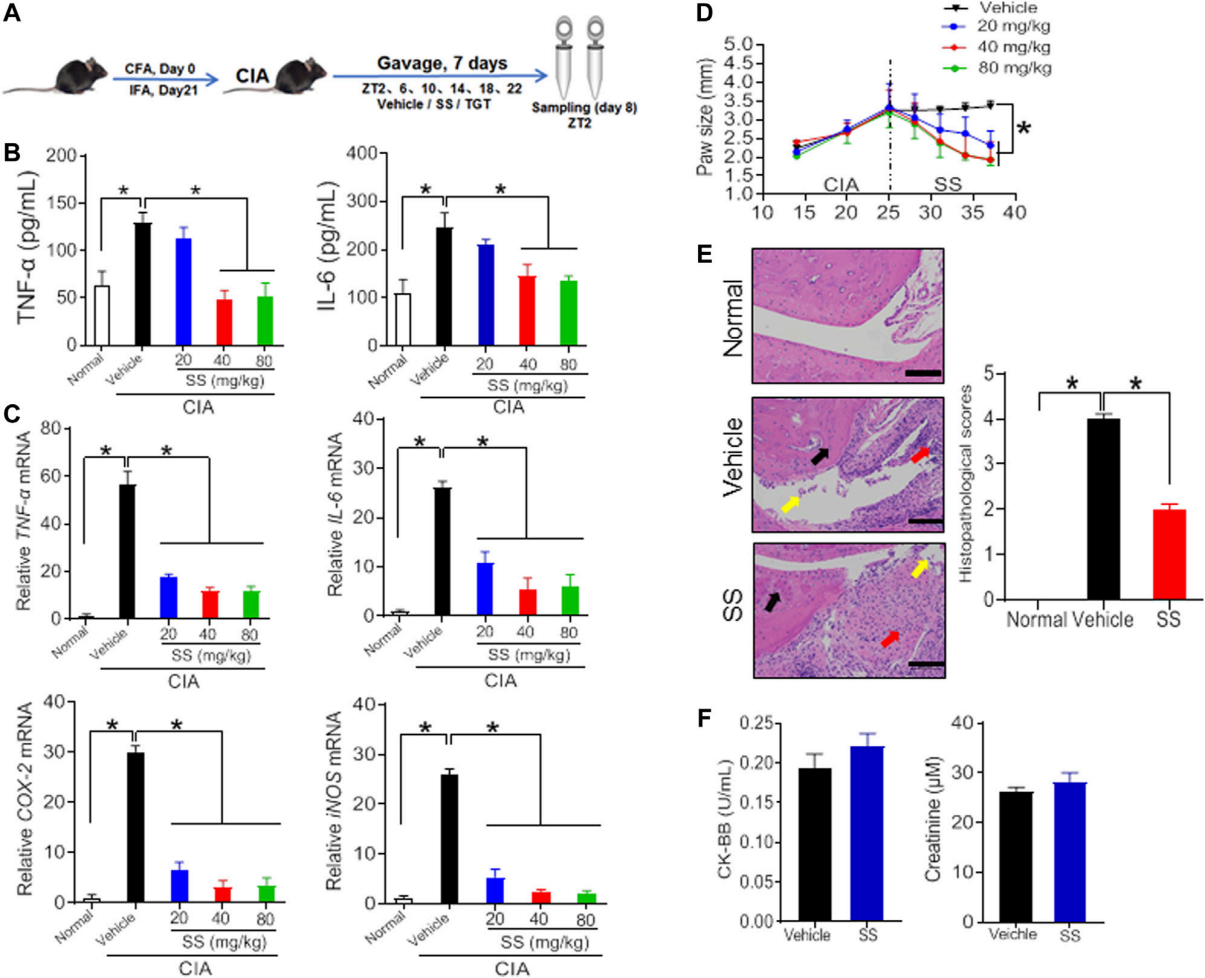
Therapeutic efficacy of SS in CIA mice **(A)** Schematic diagram for the experimental protocol **(B)** Plasma TNF-α and IL-6 levels in CIA mice after gavage with 20, 40, and 80 mg/kg SS extract or vehicle for 1 week. Data are mean ± SD (*n* = 5). *p < 0.05 (one-way ANOVA with Bonferroni *post hoc* test) **(C)** mRNA levels of *TNF-*α, *IL-6, COX-2*, and *iNOS* in CIA mice after gavage with 20, 40, and 80 mg/kg SS extract or vehicle for 1 week. Data are mean ± SD (*n* = 5). *p < 0.05 (one-way ANOVA with Bonferroni *post hoc* test) **(D)** Paw sizes in CIA mice before and after gavage of SS extract. Data are mean ± SD (*n* = 5). *p < 0.05 (one-way ANOVA with Bonferroni *post hoc* test) **(E)** H&E staining and histopathological scores. Data are mean ± SD (*n* = 5). *p < 0.05 (*t* test) **(F)** Plasma CK-BB and creatinine levels in normal mice treated with SS extract (40 mg/kg). Data are mean ± SD (*n* = 5).

**FIGURE 2:**
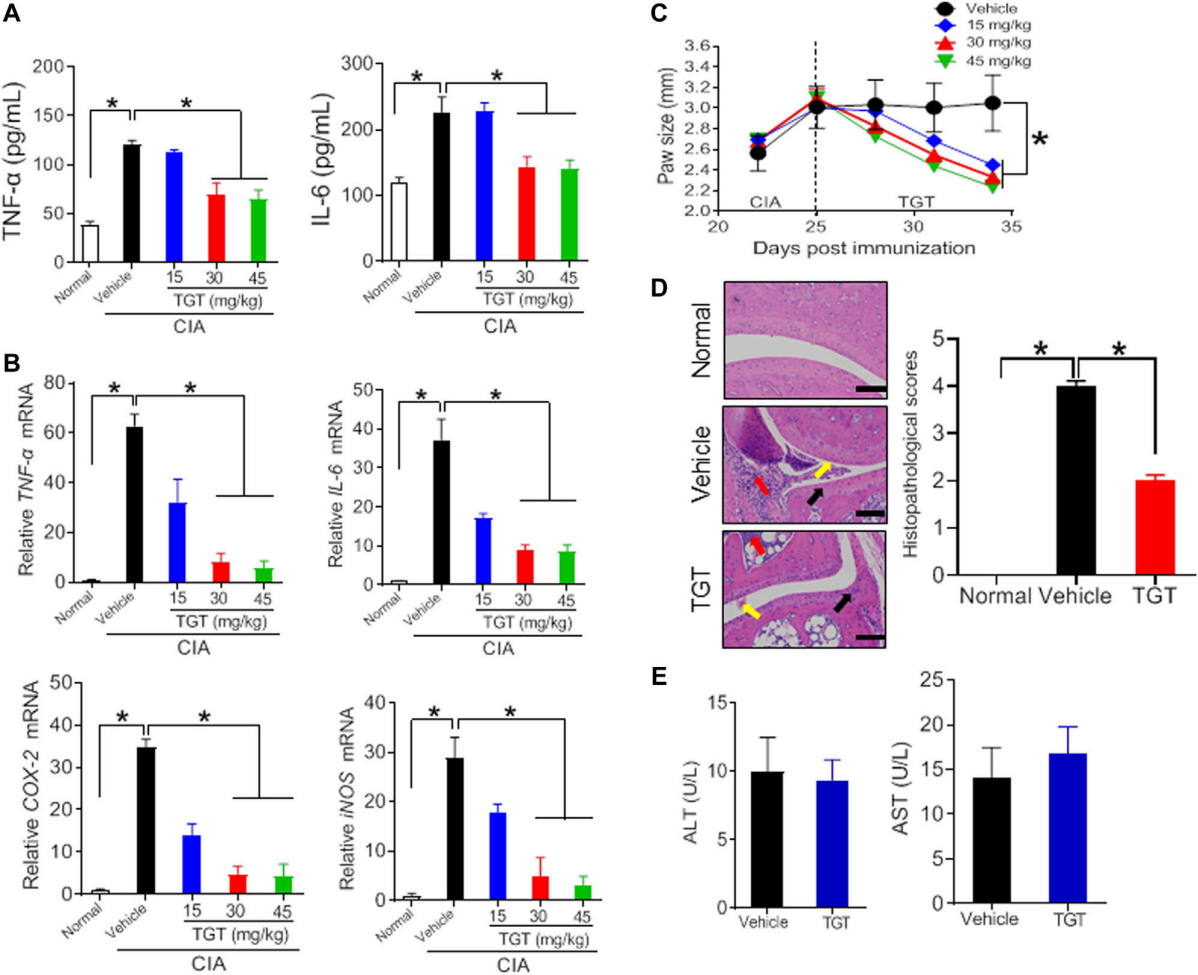
Therapeutic efficacy of TGT in CIA mice **(A)** Plasma TNF-α and IL-6 levels in CIA mice after gavage with 15, 30, and 45 mg/kg TGT suspension or vehicle for 1 week. Data are mean ± SD (*n* = 5). *p < 0.05 (one-way ANOVA with Bonferroni *post hoc* test) **(B)** mRNA levels of *TNF*-α, *IL-6, COX-2*, and *iNOS* in CIA mice after gavage with 15, 30, and 45 mg/kg TGT suspension or vehicle for 1 week. Data are mean ± SD (*n* = 5). *p < 0.05 (one-way ANOVA with Bonferroni *post hoc* test) **(C)** Paw sizes in CIA mice before and after gavage of TGT. Data are mean ± SD (*n* = 5). *p < 0.05 (one-way ANOVA with Bonferroni *post hoc* test) **(D)** H&E staining and histopathological scores. Data are mean ± SD (*n* = 5). *p < 0.05 (*t* test) **(E)** Plasma ALT and AST levels in normal mice treated with TGT (30 mg/kg). Data are mean ± SD (*n* = 5).

Collagen-induced arthritis (CIA) was established in mice as previously described (Figure 1A) (Inglis et al., 2008). As expected, CIA mice showed much higher production of TNF-α and IL-6 as well as higher mRNA expression levels of *TN*F-α, *IL-6*, *COX-2*, and *iNOS* as compared to normal mice (Figures 1B,C, 2A,B; Supplementary Table S2). In addition, H&E staining showed extensive infiltration of inflammatory cells in the synovial membranes in the joints of CIA mice and severe cartilage destruction (Figures 1E, 2D). Interestingly, SS dose-dependently reduced the production of TNF-α and IL-6 in CIA mice (Figure 1B). Furthermore, SS down-regulated the inflammatory factors *TN*F-α, *IL-6, COX-2* and *iNOS* in a dose-dependent manner (Figure 1C; Supplementary Table S2). SS also significantly ameliorated paw swelling in CIA mice (Figure 1D). In addition, SS-treated mice showed reduced infiltration of inflammatory cells and attenuated cartilage destruction according to histopathological examinations (Figure 1E). Likewise, TGT dose-dependently reduced the production of TNF-α and IL-6, and decreased the mRNA levels of TNF-α, *IL-6, COX-2* and *iNOS* in CIA mice (Figures 2A,B; Supplementary Table S2). In the meantime, TGT-treated mice showed reduced paw swelling, decreased inflammatory cell infiltration, and attenuated cartilage destruction (Figures 2C,D). These data clearly indicated anti-arthritis effects of both SS and TGT in mice. It was noteworthy that we did not observe changes in CK-BB and creatinine (respective biomarkers for neurotoxicity and nephrotoxicity) in SS (40 mg/kg)-treated mice or changes in plasma ALT and AST (two indexes of hepatotoxicity) in TGT (30 mg/kg)-treated mice (Figures 1F, 2E) (Zhao et al., 2020a; Gao et al., 2021).

### SS and TGT Efficacy Depend on Dosing Time in CIA Mice

**FIGURE 3:**
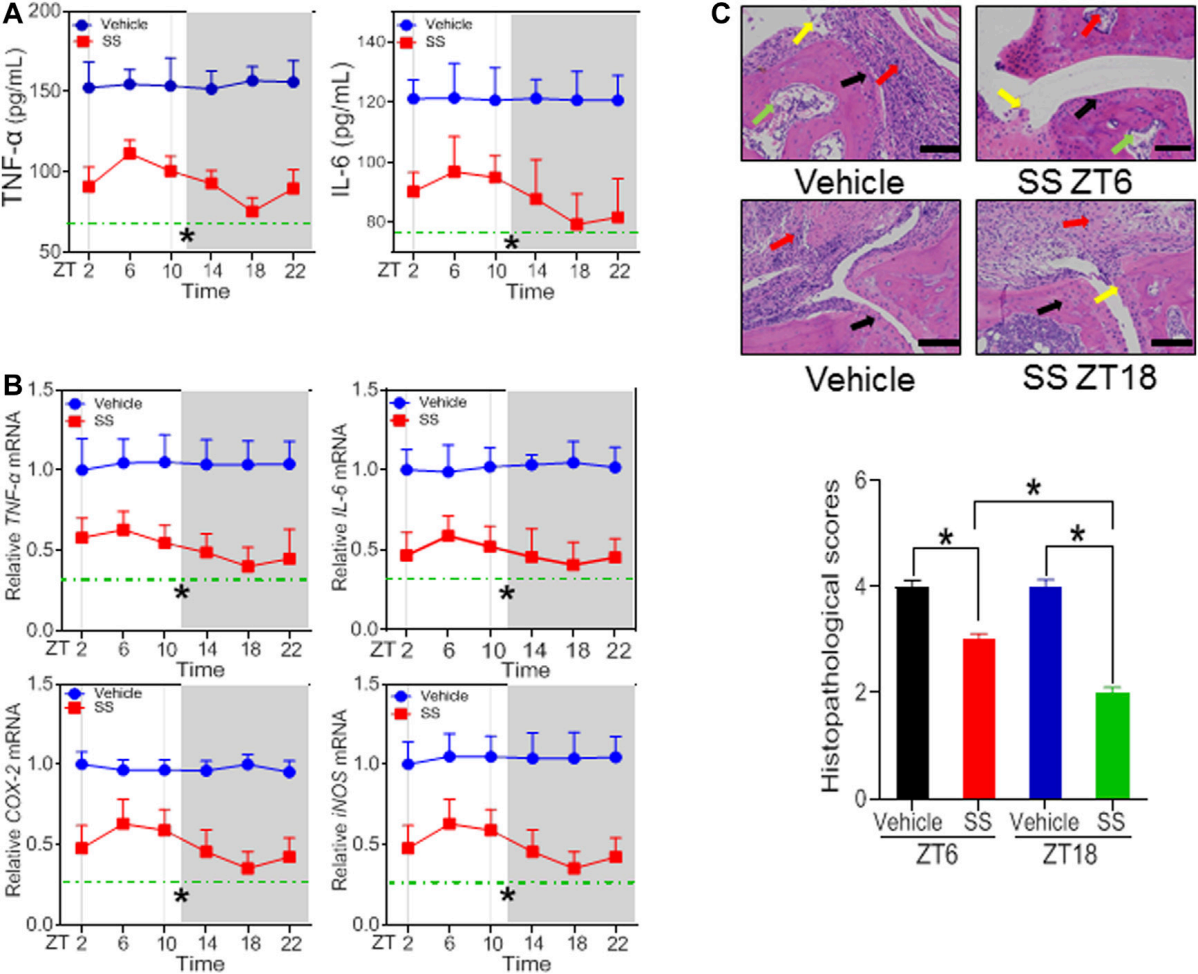
SS efficacy depends on dosing time in the CIA mice **(A)** Plasma TNF-α and IL-6 levels in SS- or vehicle-treated CIA mice at six circadian time points **(B)** mRNA levels of *TNF*-α, *IL-6, COX-2*, and *iNOS* in SS- or vehicle-treated CIA mice at six circadian time points **(C)** H&E staining and histopathological scores. Data are mean ± SD (*n* = 5). *p < 0.05 (two-way ANOVA with Bonferroni *post hoc* test).

**FIGURE 4:**
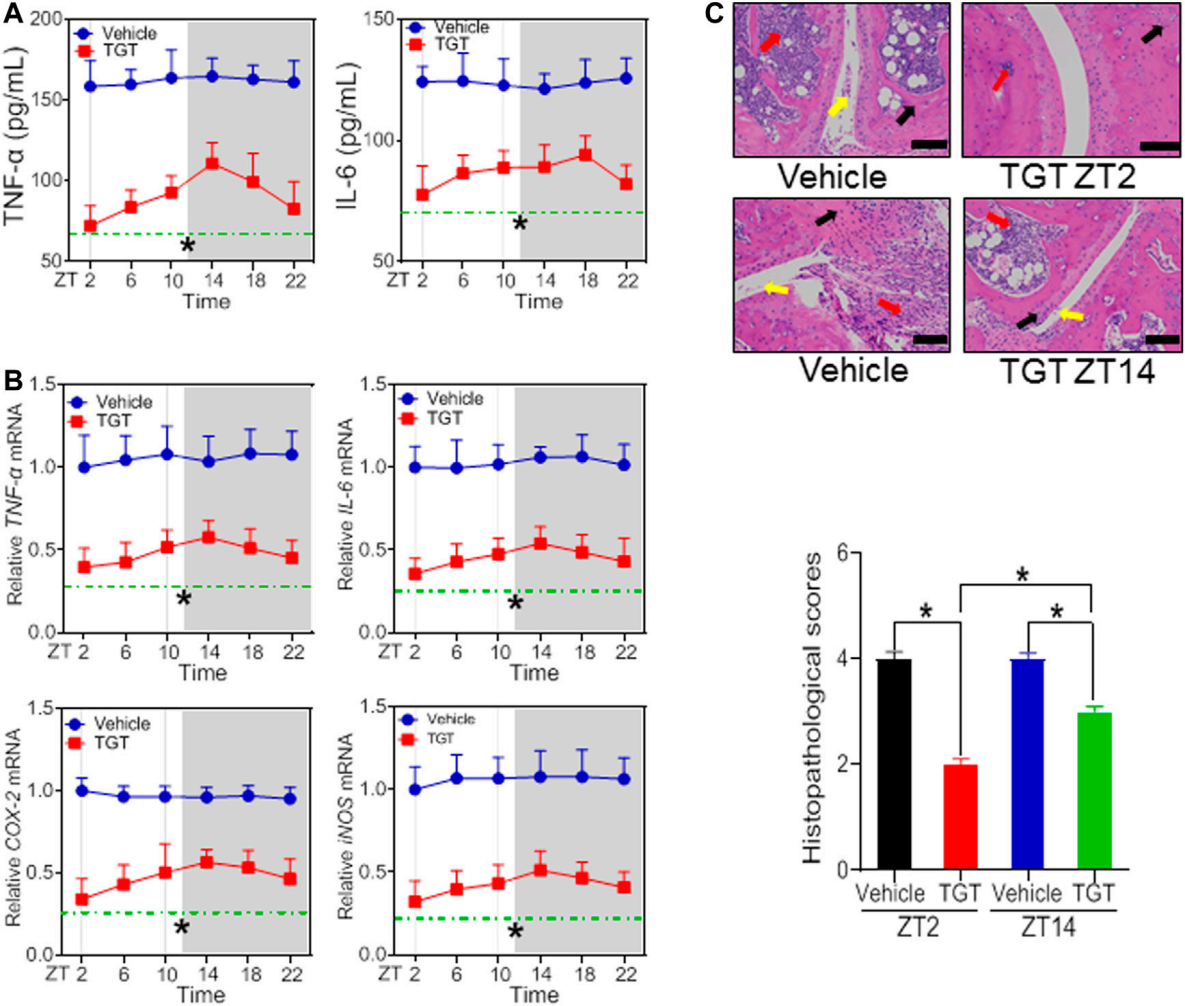
TGT efficacy depends on dosing time in the CIA mice **(A)** Plasma TNF-α and IL-6 levels in TGT- or vehicle-treated CIA mice at six circadian time points **(B)** mRNA levels of *TN*F-α, *IL-6, COX-2*, and *iNOS* in TGT- or vehicle-treated CIA mice at six circadian time points **(C)** H&E staining and histopathological scores. Data are mean ± SD (*n* = 5). *p < 0.05 (two-way ANOVA with Bonferroni *post hoc* test).

According to the dose-response experiments (Figures 1, 2), a dose of 40 mg/kg for SS and a dose of 30 mg/kg for TGT were selected to assess dosing time-dependent efficacy against CIA in mice. CIA mice were treated with SS or TGT at each of six circadian time points (ZT2, ZT6, ZT10, ZT14, ZT18, and ZT22). The therapeutic efficacy of SS and TGT against CIA displayed significant circadian rhythms (Figures 3, 4). SS efficacy was the best when drug was dosed at ZT18, and was poorer when drug was dosed at other times (particularly at ZT6, Figure 3). To be specific, ZT18 dosing generated lower levels of inflammatory factors (*TNF*-α*, IL-6, COX-2*, and *iNOS*) compared to drug dosing at other times such as ZT6 (Figures 3A,B; Supplementary Table S3). Supporting this, ZT18-treated mice showed more significantly attenuated arthritis as compared to ZT6-treated mice according to histopathological examinations (Figure 3C). As for TGT, the efficacy was better when drug was dosed at ZT2, and was poorer when drug was dosed at other times, particularly at ZT14 (Figure 4). This was accompanied by lower levels of inflammatory factors (*TNF*-α, *IL-6, COX-2*, and *iNOS*) and a smaller histopathological score at ZT2 than at ZT14 (Figures 4A–C; Supplementary Table S3). Taken together, both SS and TGT showed dosing time-dependency of anti-arthritis effects in CIA mice.

### Dosing Time-dependent Pharmacokinetics of SS and TGT

**FIGURE 5:**
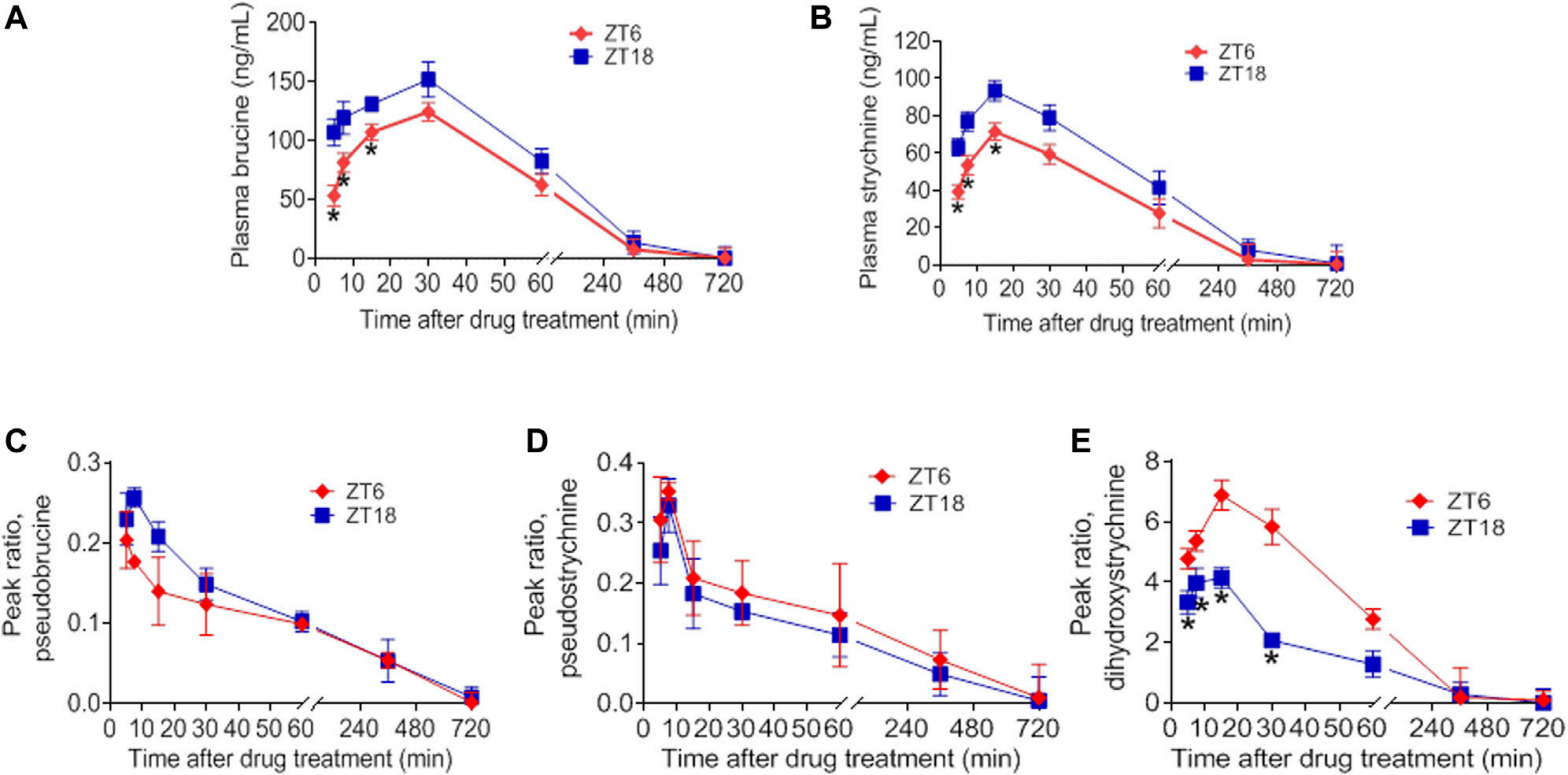
Dosing time-dependent pharmacokinetics of SS. Plasma concentration-time curve for **(A)** brucine **(B)** strychnine **(C)** pseudobrucine **(D)** pseudostrychnine, and **(E)** dihydroxystrychnine in CIA mice after gavage of SS extract (40 mg/kg) at ZT6 or ZT18. Data are mean ± SD (*n* = 5). *p < 0.05 (*t* test).

**FIGURE 6:**
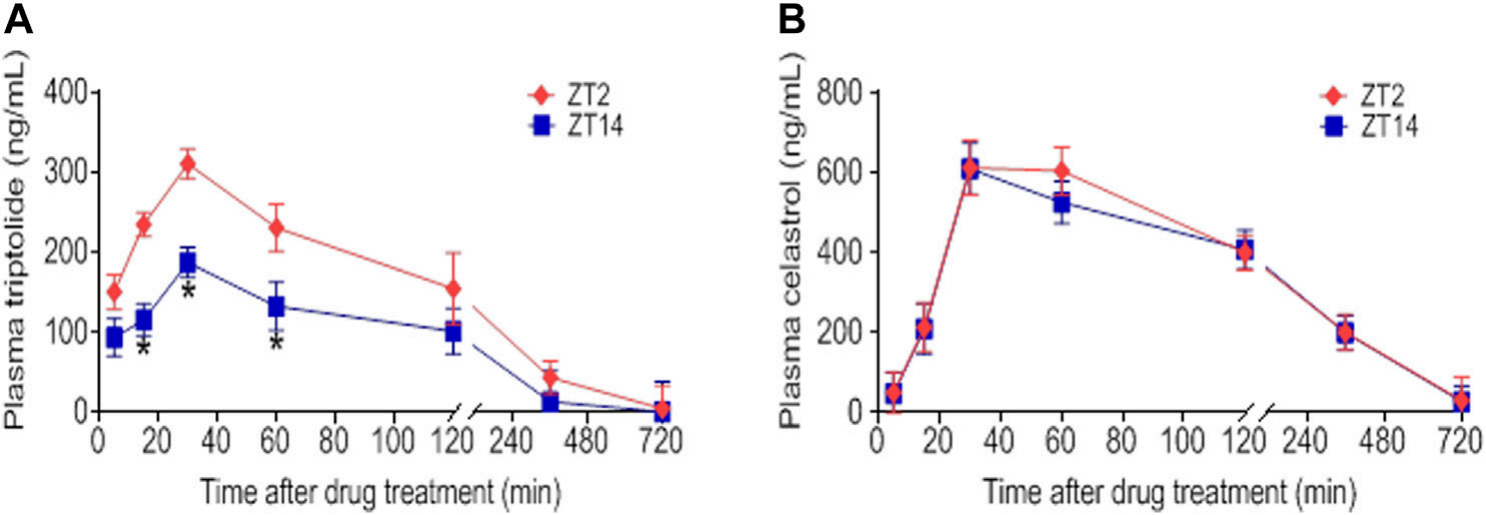
Dosing time-dependent pharmacokinetics of TGT. Plasma concentration-time curve for **(A)** triptolide and **(B)** celastrol in CIA mice after gavage of TGT suspension (30 mg/kg) at ZT2 or ZT14. Data are mean ± SD (*n* = 5). *p < 0.05 (*t* test).

Our previous studies have shown that the time-varying pharmacokinetics in normal mice contributes to chronotoxicity of SS and TGT at a toxic dose (Zhao et al., 2020b; Gao et al., 2021). Since pharmacokinetics can be a determinant of both drug efficacy and toxicity, it is of interest to test whether the pharmacokinetics of SS and TGT in CIA mice are circadian-dependent or not at a therapeutic dose. CIA mice were treated with SS (40 mg/kg) at ZT6 (corresponding to low drug efficacy) or ZT18 (corresponding to high drug efficacy), and with TGT (30 mg/kg) at ZT2 (corresponding to high drug efficacy) or ZT14 (corresponding to low drug efficacy) (Figures 5, 6). Pharmacokinetic analysis of SS focused on four constituents (i.e., brucine, strychnine, pseudostrychnine, and pseudobrucine) and one metabolite (dihydroxystrychnine from strychnine) that can be quantified as noted previously (Zhao et al., 2020a; Gao et al., 2021). Plasma levels of brucine and strychnine after SS dosing at ZT18 were significantly higher than those after drug dosing at ZT6, particularly in the first 30 min (Figures 5A,B). Accordingly, the AUC values of brucine and strychnine were higher at ZT18 than at ZT6 (Table 2). By contrast, pharmacokinetic behaviors of other two constituents (pseudostrychnine and pseudobrucine) showed no differences between two circadian time points (Figures 5C,D). Furthermore, a significant dosing-time effect was observed for strychnine metabolism (i.e., formation of dihydroxystrychnine) (Figure 5E). Plasma dihydroxystrychnine levels were higher at dosing time of ZT6 than at ZT18 (Figure 5E). Lower metabolite formation may account for higher exposure of strychnine at ZT18 (Figures 5B,E).

**TABLE 2:** Pharmacokinetic parameters for strychnine and brucine in CIA mice after oral gavage of SS extract (40 mg/kg).

Parameter	Unit	Strychnine		Brucine	
		ZT6	ZT18	ZT6	ZT18
t_1/2_	Min	47.3 ± 2.18	55.7 ± 1.98	57.8 ± 3.69	147.3 ± 9.32*
C_max_	ng/ml	66.8 ± 2.84	75.9 ± 1.65*	151.32 ± 14.21	188.36 ± 10.98
AUC_0-t_	ng*min/ml	4,339 ± 88.5	4,766 ± 61.27*	9,006 ± 249	11,923 ± 498*

* p < 0.05 vs. ZT6.

Triptolide, celastrol, tripdiolide, wilfordine, and wilfortrine are five main putative active constituents of TGT (Bao and Dai, 2011). It was hypothesized that the chronoefficacy of TGT might be associated with time-varying exposure of these compounds, which were then the focuses of pharmacokinetic analysis for TGT. We found that TGT dosing at ZT2 generated higher plasma concentrations of triptolide as compared to ZT14 dosing (Figure 6A). Consequently, the systemic exposure (reflected by AUC) of triptolide at ZT2 was higher than that at ZT14 (Table 3). In contrast, plasma concentrations of celastrol showed no significant differences between two dosing times (Figure 6B). It was noteworthy that we were unable to analyze pharmacokinetic behaviors of the other three constitutes tripdiolide, wilfordine and wilfortrine because their plasma levels were below the lower limit of quantification. Taken together, time-varying exposure of triptolide may account for dosing time-dependent efficacy of TGT.

**TABLE 3:** Pharmacokinetic parameters for triptolide in CIA mice after oral gavage of TGT suspension (30 mg/kg).

Parameter	Unit	Triptolide	
		ZT2	ZT14
t_1/2_	Min	153.2 ± 49.58	208.3 ± 21.31
C_max_	ng/ml	293.5 ± 18.95	194.3 ± 10.12*
AUC_0-t_	ng*min/ml	41.2 ± 5.02	19.1 ± 1.988*

* p < 0.05 vs. ZT2.

### Anti-Arthritis Effects of Brucine, Strychine and Triptolide *in vitro*

**FIGURE 7:**
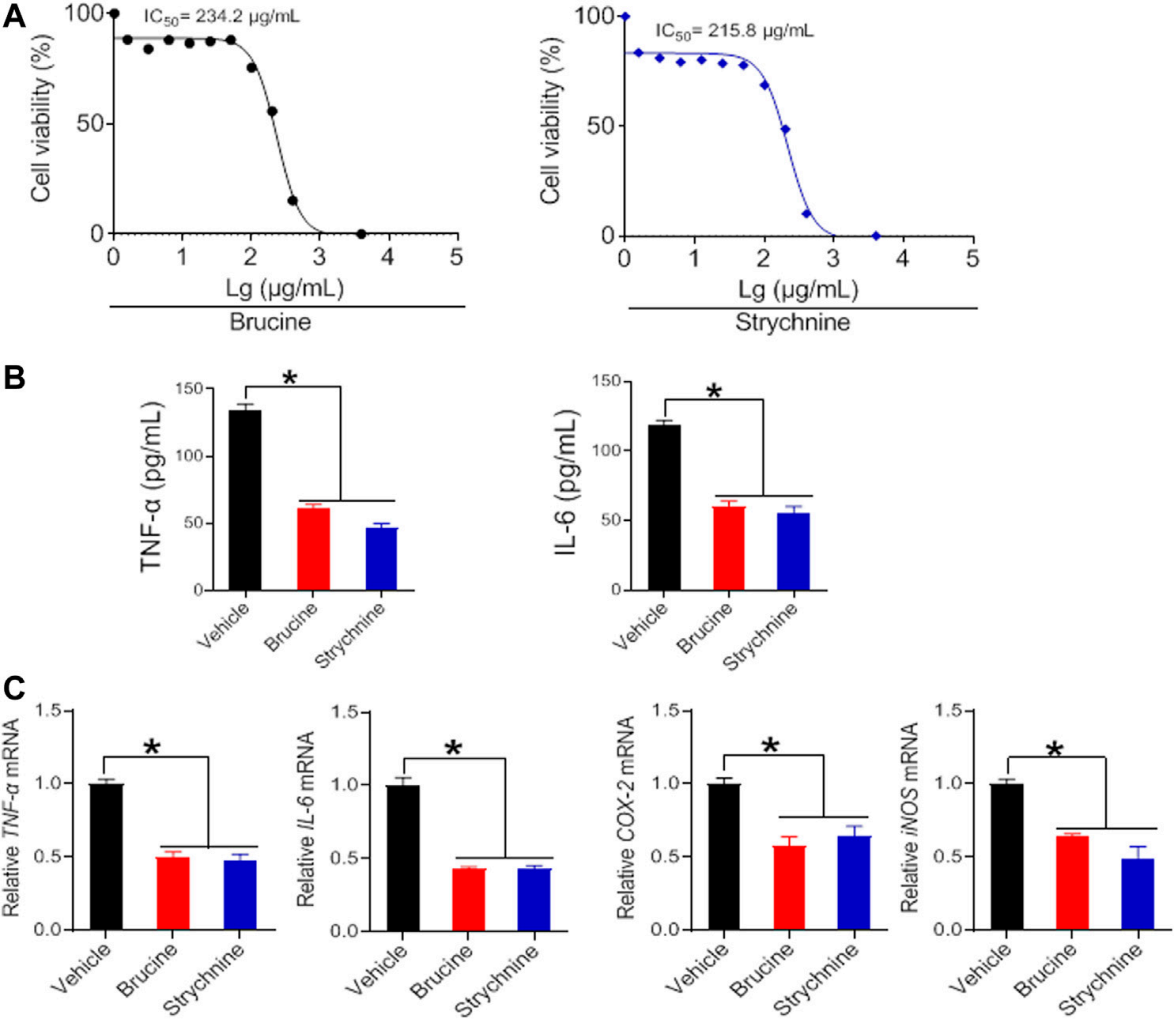
Brucine and strychine inhibit *TNF*-α, *IL-6*, *COX-2*, and iNOS expression in FLS cells **(A)** Effects of brucine and strychnine at indicated concentrations on the viability of FLS cells **(B)** Effects of brucine and strychnine (50 μg/ml) on the production of TNF-α and IL-6 in FLS cells. Data are mean ± SD (*n* = 5). *p < 0.05 (*t* test) **(C)** Effects of brucine and strychnine (50 μg/ml) on *TNF*-α, *IL-6, COX-2,* and *iNOS* mRNAs in FLS cells. Data are mean ± SD (*n* = 5). *p < 0.05 (*t* test).

**FIGURE 8:**
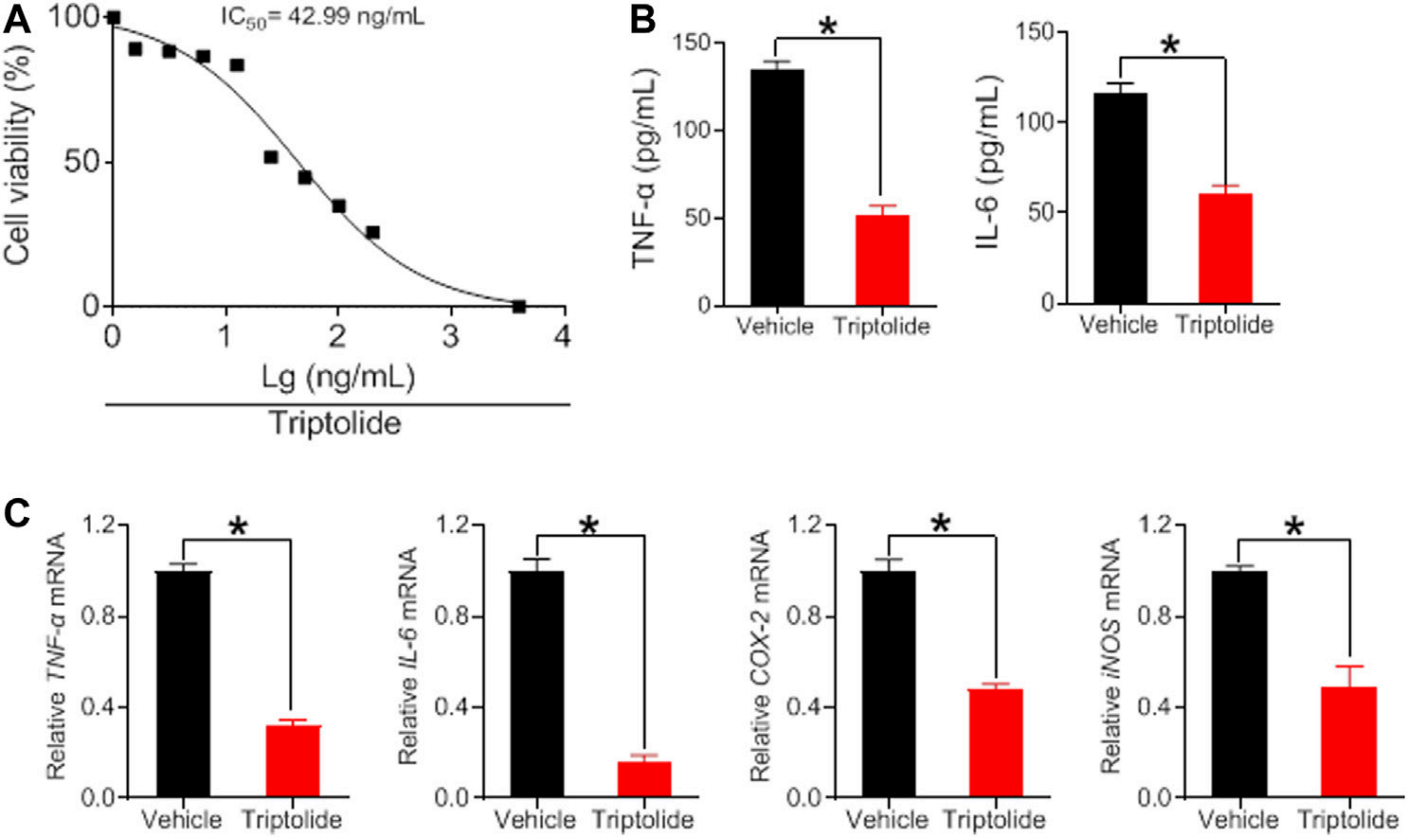
Triptolide inhibits *TNF*-α, *IL-6*, *COX-*2, and *iNOS* expression in FLS cells **(A)** Effects of triptolide at indicated concentrations on the viability in FLS cells **(B)** Effects of triptolide (6.25 ng/ml) on the production of TNF-α and IL-6 in FLS cells. Data are mean ± SD (*n* = 5). *p < 0.05 (*t*-test) **(C)** Effects of triptolide (6.25 ng/ml) on *TNF-*α*, IL-6, COX-2*, and *iNOS* mRNAs in FLS cells. Data are mean ± SD (*n* = 5). *p < 0.05 (*t* test).

To verify whether the herbal constituents, brucine, strychine and triptolide possess an anti-arthritis activity, we examined their effects on the proliferation of FLS and the expression of the inflammatory factors *TNF*-α, I*L-6, COX-2*, and *iNOS*. FLS cells were isolated from the joints of CIA mice, and treated with different concentrations of brucine, strychine or triptolide. We found that brucine, strychnine and triptolide significantly inhibited the proliferation of FLS cells with low IC_50_ values (234.2, 215.8 μg/ml and 42.99 ng/ml for brucine, strychnine and triptolide, respectively) (Figures 7A, 8A). Furthermore, TNF-α and IL-6 production were significantly reduced in brucine-, strychine-, and triptolide-treated FLS (Figures 7B, 8B). In addition, brucine, strychine and triptolide significantly inhibited the expression of arthritis-related inflammatory factors (i.e., *TNF*-α, IL-6, *COX-2*, and *iNOS*) (Figures 7C, 8C). Taken together, brucine and strychine (two putative active constitutes of SS) as well as triptolide (a putative active constitute of TGT) can inhibit the proliferation of FLS and the expression of key inflammatory factors, suggesting that they do possess an anti-arthritis activity.

## Discussion

In this study, we have observed time-varying efficacy (chronoefficacy) of two herbal medicines SS and TGT against CIA in mice (Figures 3, 4). The chronoefficacy of SS and TGT were respectively associated with circadian pharmacokinetics of strychnine/brucine (two ingredients of SS) and triptolide (an ingredient of TGT) (Figures 5, 6). Specifically, SS dosed at ZT18 were more effective in protecting against CIA than drug dosed at ZT6 (Figure 3, lower levels of inflammatory factors at ZT18 than that at ZT6). This was accompanied by higher exposures of strychnine and brucine in ZT18-treated CIA mice than in ZT6-treated CIA mice at a therapeutic dose (Figure 5). As for TGT, drug dosing at ZT2 showed superior efficacy against CIA as compared to drug dosing at ZT14 (Figure 4). Consistently, ZT2 dosing generated a higher exposure of triptolide as compared to ZT14 in CIA mice (Figure 6). Moreover, strychnine, brucine and triptolide possessed an anti-arthritis activity as they inhibited the proliferation of FLSs and reduced the production of TNF-α/IL-6 and mRNAs levels of *TNF*-α, *IL-6, COX-2*, and *iNOS* (Figures 7, 8). Thus, these compounds were potential active ingredients of SS and TGT against CIA. We therefore propose that circadian pharmacokinetics of active ingredients is responsible for chronoefficacy of SS and TGT against rheumatoid arthritis. Our findings have implications for improving treatment outcomes of SS and TGT *via* timed delivery.

Rheumatoid arthritis begins with chronic systemic inflammation and angiogenesis in the synovial cavity, which will progress to the formation of synovial membrane and to osteoporosis, eventually leading to destruction of joints and bone absorption (Karmakar et al., 2010). Synovial inflammation disrupts the balance between bone resorption by osteoclast mediators and osteoblast-mediated bone formation at the earliest stage of rheumatoid arthritis (Karmakar et al., 2010). Therefore, blocking the inflammatory cascade in the early stage of rheumatoid arthritis can partially reverse the aggressive bone damage and prevent the destructive progression (Li et al., 2015). Diurnal variations in the flare of symptoms of rheumatoid arthritis have been recognized for a long time. Clinical studies in 1960s have documented an increased joint stiffness in patients of rheumatoid arthritis in the morning (Gibbs and Ray., 2013). Supporting this, the levels of inflammatory factors such as IL-6 (the marker of rheumatoid arthritis) display diurnal oscillations with a peak level in the morning (Ingpen, 1968). Notably, circadian variation in the severity of disease is a potential factor contributing to time-varying drug effects (Zhao et al., 2020a). It was thus necessary to minimize this confounding effect on the chronoefficacy of SS and TGT as the efficacy against arthritis was mainly measured by changes in the levels of inflammatory factors. To this end, we sampled SS- and TGT-treated CIA mice at the same circadian time point (i.e., ZT2) after time-dependent treatments. By doing this, effects of circadian flare of symptoms (circadian time-varying inflammatory factors) on chronoefficacy of SS and TGT can be excluded.

Cyp3a11, a member of cytochrome P450 family of enzymes, plays a major role in the drug metabolism and detoxification (Lin et al., 2019a, Lin et al., 2019b). In a previous study, diurnal expression of Cyp3a11 contributed to time-varying pharmacokinetics and toxicity of triptolide (a main toxic ingredient of TGT) (Lin et al., 2019a). In addition, circadian Cyp3a11 metabolism is a potential mechanism accounting for the chronotoxicity of Tripterygium wilfordii (Zhao et al., 2020b). Since circadian metabolism and pharmacokinetics can be a cause of both chronotoxicity and chronoefficacy of drugs, we propose that pharmacokinetics-based chronoefficacy of TGT is regulated by CYP3A11. In fact, diurnal expression of Cyp3a11 (a lower expression at ZT2 than at ZT14) may lead to a higher efficacy of TGT at ZT2 than at ZT14 due to a lower metabolic/clearance ability at ZT2 than at ZT14 (Figure 4).

To assess the therapeutic efficacy of SS and TGT against rheumatoid arthritis, we established mouse model with type II collagen (CII)-induced arthritis (CIA). CIA is one of the widely used animal models sharing many pathological and histological similarities with rheumatoid arthritis in humans (Nandakumar and Holmdahl, 2007). Chronic inflammation of joints is a main characteristic of rheumatoid arthritis and CIA (Seeling et al., 2013). It has been reported that autoantibodies to CII and CII-specific T-cell response play an important role in the pathogenesis of CIA (Seeling et al., 2013). Intriguingly, CII is the major protein constituent of cartilage in diarthrodial joint which is the predominant site of inflammation in rheumatoid arthritis (Myers et al., 1997). In addition, both rheumatoid arthritis and CIA are characterized by an intense synovitis and ensuing erosions of cartilage and subchondral bone by a pannus-like tissue (Myers et al., 1997). Infiltration of inflammatory cells and chemokine production are two main features of synovitis (Yoshimura et al., 2014). Inflammatory cells migrated into the joints produce several proinflammatory cytokines including IL-6 and IL-1 that induce osteoclastogenesis by acting on osteoclast precursor and lead to bone erosion (Yoshimura et al., 2014).

Although SS and TGT are therapeutically beneficial in the treatment of rheumatoid arthritis, their toxicities to certain organs have been noted (Zhao et al., 2020a; Gao et al., 2021). SS mainly causes injuries in the kidney and brain, thereby generating nephrotoxicity and neurotoxicity (Gao et al., 2021). By contrast, TGT mainly causes damages in the liver, thereby resulting in hepatotoxicity (Zhao et al., 2020a). Thus, it was necessary to determine the toxicities of SS and TGT at the tested (therapeutic) doses. The nephrotoxicity and neurotoxicity were respectively determined by measuring plasma creatinine (an index for measurement of renal function) and CK-BB (a biomarker of neurotoxicity), while the hepatotoxicity was evaluated by measuring plasma AST and ALT (Zhao et al., 2020b; Gao et al., 2021). We did not observe changes in CK-BB and creatinine in SS (40 mg/kg)-treated mice or changes in plasma ALT and AST in TGT (30 mg/kg)-treated mice (Figures 1F, 2E ). Therefore, SS and TGT are two superior herbal medicines for treating rheumatoid arthritis in terms of safety.

FLS cell is a major component of rheumatoid pannus (a complication of rheumatoid arthritis) and plays a key role in its formation (Bottini and Firestein, 2013). FLS cells ensure the structural integrity of a normally organized synovial lining and secrete hyaluronic acid and lubricin (two important constituents of synovial fluid that is responsible for lubricating the joint) in healthy individuals (Kiener et al., 2010; Filer, 2013). However, FLS cells have an increased ability to invade periarticular tissues including bone and cartilage after acquiring an aggressive phenotype (Kiener et al., 2010). These stimulated cells produce several mediators that induce angiogenesis. Therefore, FLS is considered to be an initiator of rheumatoid arthritis (Aupperle et al., 1998; Roccaro et al., 2005). In our study, FLS cells were isolated from CIA mice. The proliferation of these cells was significantly faster than cells derived from normal mice (Aupperle et al., 1998). However, brucine, strychine and triptolide significantly inhibited the proliferation of FLS and the expression of inflammatory factors (i.e., TNF-α, IL-6, COX-2, and iNOS) (Figures 7, 8). The data strongly suggested anti-arthritis effects of brucine, strychine and triptolide, and supported these compounds as active constituents of SS and TGT against rheumatoid arthritis.

It is noteworthy that CIA was assessed herein based on the levels of the inflammatory factors TNF-α, IL-6, COX-2, and iNOS. IL-6 and TNF-α are proinflammatory cytokines whose elevations have been associated with pathologies of rheumatoid arthritis (Wilburn et al., 2021). Meanwhile, elevated TNF-α and IL-6 stimulate the expression of rheumatoid arthritis-related proteins such as C-reactive protein (Inanc et al., 2021). COX-2 plays an important role in articular cartilage disorders. It enhances inflammatory cytokine-induced metabolic imbalance of cartilage proteoglycans, irreversibly promoting and aggravating arthritis (Li et al., 2015). iNOS is a key enzyme responsible for regulating the physiological and pathological effects of NO (Bao and Dai, 2011). Elevated NO level in rheumatoid arthritis has been linked to increases in the inflammatory markers (Garg et al., 2017). Additionally, production of NO is abnormally increased in rheumatoid arthritis patients (Bao and Dai, 2011).

In conclusion, the herbal medicines SS and TGT display chronoefficacy against rheumatoid arthritis in mice, that is attributed to circadian pharmacokinetics of main active ingredients. Our findings have implications for improving treatment outcomes of SS and TGT *via* timed delivery.

## Data Availability

The raw data supporting the conclusions of this article will be made available by the authors, without undue reservation, to any qualified researcher.
