# Supplementary material for: Pharmacokinetics-Based Chronoefficacy of Semen Strychni and Tripterygium Glycoside Tablet Against Rheumatoid Arthritis
Source: Front Pharmacol. 2021 May 24;12:673263. doi: 10.3389/fphar.2021.673263 (PMC8181759; doi:10.3389/fphar.2021.673263)
Supplement: Supplementary file 1 [file DataSheet1.docx]

**Supplementary materials**

**Table S1: Main active compounds of SS and TGT.**

|  | **Compounds** | **RT [min]** | **[M+H]^+^** | **Molecular formula** | **Extract content (mg/g)** |
| --- | --- | --- | --- | --- | --- |
| **SS** | Strychnine | 2.70 | 335.18 | C_21_H_22_N_2_O_2_ | 4.53 ± 0.25 |
|  | Brucine | 2.68 | 395.20 | C_23_H_26_N_2_O_4_ | 2.73 ± 0.13 |
| **TGT** | Triptolide | 1.95 | 361.17 | C_20_H_24_O_6_ | 1.08 ± 0.09 |
|  | Celastrol | 3.97 | 451.29 | C_29_H_38_O_4_ | 28.64 ± 1.86 |

**Table S2: Raw data on relative expression of inflammatory biomarkers (Figures 1C & 2B)**

|  | **SS** | **Mean** | **SD** | **TGT** | **Mean** | **SD** |
| --- | --- | --- | --- | --- | --- | --- |
| ***TNF-α*** | Normal | 1.00 | 1.13 | Normal | 1.00 | 0.20 |
|  | Vehicle | 56.44 | 5.68 | Vehicle | 62.79 | 4.43 |
|  | 20 mg/kg | 17.81 | 1.10 | 15 mg/kg | 32.24 | 9.19 |
|  | 40 mg/kg | 11.88 | 1.49 | 30 mg/kg | 8.17 | 3.57 |
|  | 80 mg/kg | 12.04 | 1.81 | 45 mg/kg | 5.81 | 2.87 |

|  | **SS** | **Mean** | **SD** | **TGT** | **Mean** | **SD** |
| --- | --- | --- | --- | --- | --- | --- |
| ***IL-6*** | Normal | 1.00 | 0.29 | Normal | 1.00 | 0.02 |
|  | Vehicle | 26.19 | 1.18 | Vehicle | 37.19 | 5.27 |
|  | 20 mg/kg | 10.79 | 2.29 | 15 mg/kg | 17.06 | 1.19 |
|  | 40 mg/kg | 5.39 | 2.33 | 30 mg/kg | 8.84 | 1.34 |
|  | 80 mg/kg | 6.10 | 2.35 | 45 mg/kg | 8.64 | 1.58 |

|  | **SS** | **Mean** | **SD** | **TGT** | **Mean** | **SD** |
| --- | --- | --- | --- | --- | --- | --- |
| ***COX-2*** | Normal | 1.00 | 0.58 | Normal | 1.00 | 0.26 |
|  | Vehicle | 30.06 | 1.18 | Vehicle | 34.80 | 1.85 |
|  | 20 mg/kg | 6.41 | 1.68 | 15 mg/kg | 13.88 | 2.76 |
|  | 40 mg/kg | 3.00 | 1.42 | 30 mg/kg | 4.76 | 1.91 |
|  | 80 mg/kg | 3.50 | 1.46 | 45 mg/kg | 4.37 | 2.74 |

|  | **SS** | **Mean** | **SD** | **TGT** | **Mean** | **SD** |
| --- | --- | --- | --- | --- | --- | --- |
| ***iNOS*** | Normal | 1.00 | 0.56 | Normal | 1.00 | 0.49 |
|  | Vehicle | 25.89 | 1.18 | Vehicle | 28.89 | 4.17 |
|  | 20 mg/kg | 5.34 | 1.59 | 15 mg/kg | 17.75 | 1.76 |
|  | 40 mg/kg | 2.22 | 0.61 | 30 mg/kg | 5.02 | 3.67 |
|  | 80 mg/kg | 2.00 | 0.60 | 45 mg/kg | 3.26 | 1.67 |

**Table S3: Raw data on relative expression of inflammatory biomarkers (Figures 3B & 4B)**

|  |  |  | **Mean** | **SD** |  |  | **Mean** | **SD** |
| --- | --- | --- | --- | --- | --- | --- | --- | --- |
| ***TNF-α*** | Vehicle | ZT2 | 1.00 | 0.20 | Vehicle | ZT2 | 1.00 | 0.20 |
|  |  | ZT6 | 1.04 | 0.15 |  | ZT6 | 1.04 | 0.15 |
|  |  | ZT10 | 1.05 | 0.17 |  | ZT10 | 1.08 | 0.17 |
|  |  | ZT14 | 1.03 | 0.15 |  | ZT14 | 1.03 | 0.15 |
|  |  | ZT18 | 1.03 | 0.15 |  | ZT18 | 1.08 | 0.15 |
|  |  | ZT22 | 1.04 | 0.14 |  | ZT22 | 1.08 | 0.14 |
|  | SS(40 mg/kg) | ZT2 | 0.58 | 0.12 | TGT(30 mg/kg) | ZT2 | 0.40 | 0.11 |
|  |  | ZT6 | 0.63 | 0.11 |  | ZT6 | 0.43 | 0.12 |
|  |  | ZT10 | 0.55 | 0.11 |  | ZT10 | 0.52 | 0.10 |
|  |  | ZT14 | 0.49 | 0.12 |  | ZT14 | 0.58 | 0.10 |
|  |  | ZT18 | 0.40 | 0.12 |  | ZT18 | 0.51 | 0.12 |
|  |  | ZT22 | 0.45 | 0.18 |  | ZT22 | 0.45 | 0.11 |

|  |  |  | **Mean** | **SD** |  |  | **Mean** | **SD** |
| --- | --- | --- | --- | --- | --- | --- | --- | --- |
| ***IL-6*** | Vehicle | ZT2 | 1.00 | 0.14 | Vehicle | ZT2 | 1.00 | 0.13 |
|  |  | ZT6 | 0.99 | 0.12 |  | ZT6 | 1.00 | 0.17 |
|  |  | ZT10 | 1.02 | 0.13 |  | ZT10 | 1.02 | 0.12 |
|  |  | ZT14 | 1.03 | 0.18 |  | ZT14 | 1.06 | 0.06 |
|  |  | ZT18 | 1.04 | 0.14 |  | ZT18 | 1.06 | 0.13 |
|  |  | ZT22 | 1.01 | 0.12 |  | ZT22 | 1.01 | 0.12 |
|  | SS(40 mg/kg) | ZT2 | 0.47 | 0.14 | TGT(30 mg/kg) | ZT2 | 0.36 | 0.09 |
|  |  | ZT6 | 0.59 | 0.12 |  | ZT6 | 0.43 | 0.11 |
|  |  | ZT10 | 0.52 | 0.13 |  | ZT10 | 0.48 | 0.10 |
|  |  | ZT14 | 0.46 | 0.18 |  | ZT14 | 0.54 | 0.10 |
|  |  | ZT18 | 0.41 | 0.14 |  | ZT18 | 0.49 | 0.11 |
|  |  | ZT22 | 0.45 | 0.12 |  | ZT22 | 0.43 | 0.14 |

|  |  |  | **Mean** | **SD** |  |  | **Mean** | **SD** |
| --- | --- | --- | --- | --- | --- | --- | --- | --- |
| ***COX-2*** | Vehicle | ZT2 | 1.00 | 0.08 | Vehicle | ZT2 | 1.00 | 0.08 |
|  |  | ZT6 | 0.96 | 0.06 |  | ZT6 | 0.96 | 0.06 |
|  |  | ZT10 | 0.96 | 0.06 |  | ZT10 | 0.96 | 0.06 |
|  |  | ZT14 | 0.96 | 0.06 |  | ZT14 | 0.96 | 0.06 |
|  |  | ZT18 | 1.00 | 0.06 |  | ZT18 | 0.97 | 0.06 |
|  |  | ZT22 | 0.95 | 0.07 |  | ZT22 | 0.95 | 0.07 |
|  | SS(40 mg/kg) | ZT2 | 0.48 | 0.14 | TGT(30 mg/kg) | ZT2 | 0.34 | 0.12 |
|  |  | ZT6 | 0.63 | 0.15 |  | ZT6 | 0.43 | 0.12 |
|  |  | ZT10 | 0.59 | 0.13 |  | ZT10 | 0.50 | 0.17 |
|  |  | ZT14 | 0.45 | 0.14 |  | ZT14 | 0.57 | 0.08 |
|  |  | ZT18 | 0.35 | 0.10 |  | ZT18 | 0.53 | 0.10 |
|  |  | ZT22 | 0.42 | 0.12 |  | ZT22 | 0.46 | 0.12 |
|  |  |  | **Mean** | **SD** |  |  | **Mean** | **SD** |
| ***iNOS*** | Vehicle | ZT2 | 1.00 | 0.14 | Vehicle | ZT2 | 1.00 | 0.14 |
|  |  | ZT6 | 1.05 | 0.14 |  | ZT6 | 1.07 | 0.14 |
|  |  | ZT10 | 1.05 | 0.13 |  | ZT10 | 1.07 | 0.13 |
|  |  | ZT14 | 1.04 | 0.16 |  | ZT14 | 1.08 | 0.16 |
|  |  | ZT18 | 1.04 | 0.16 |  | ZT18 | 1.08 | 0.16 |
|  |  | ZT22 | 1.04 | 0.13 |  | ZT22 | 1.06 | 0.13 |
|  | SS(40 mg/kg) | ZT2 | 0.48 | 0.14 | TGT(30 mg/kg) | ZT2 | 0.32 | 0.13 |
|  |  | ZT6 | 0.63 | 0.15 |  | ZT6 | 0.40 | 0.11 |
|  |  | ZT10 | 0.59 | 0.13 |  | ZT10 | 0.43 | 0.11 |
|  |  | ZT14 | 0.45 | 0.14 |  | ZT14 | 0.51 | 0.12 |
|  |  | ZT18 | 0.35 | 0.10 |  | ZT18 | 0.47 | 0.10 |
|  |  | ZT22 | 0.42 | 0.12 |  | ZT22 | 0.41 | 0.09 |

**Figure S1: Representative chromatograms of** **strychnine, brucine and their metabolites in SS exaction derived from UPLC-QTOF/MS analysis. The insert shows chemical structure.**


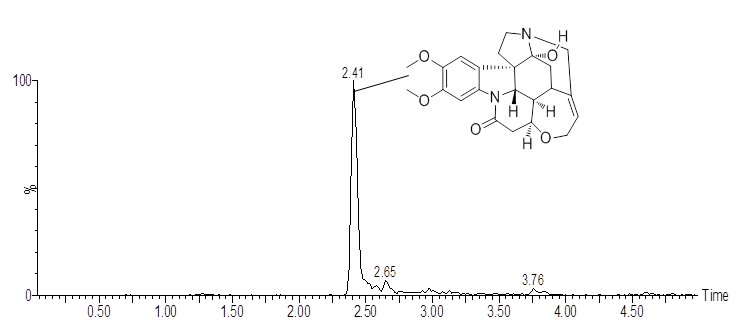

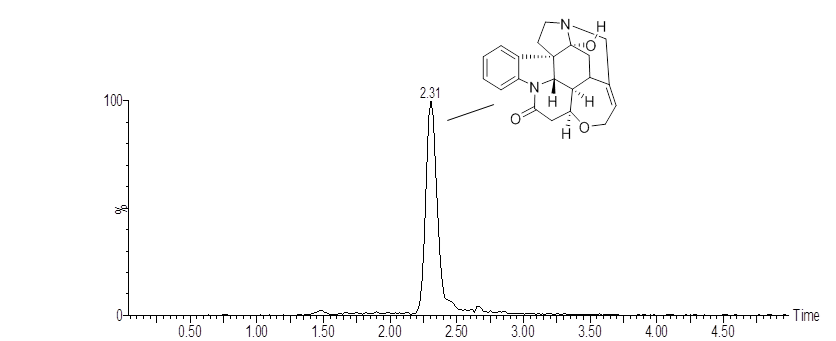

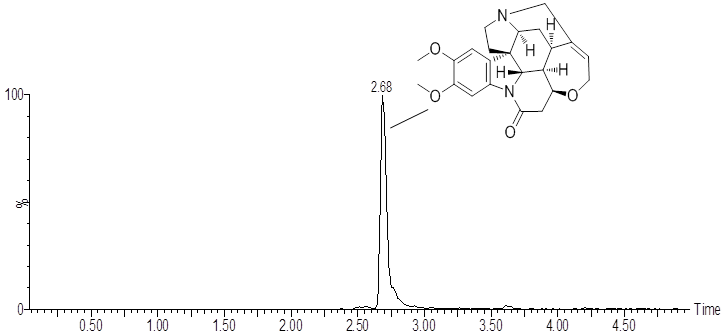

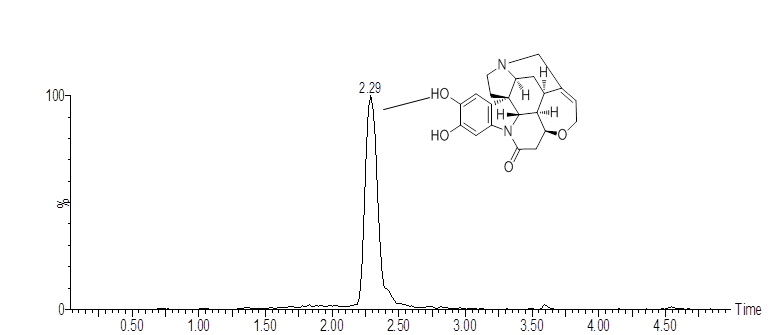
**
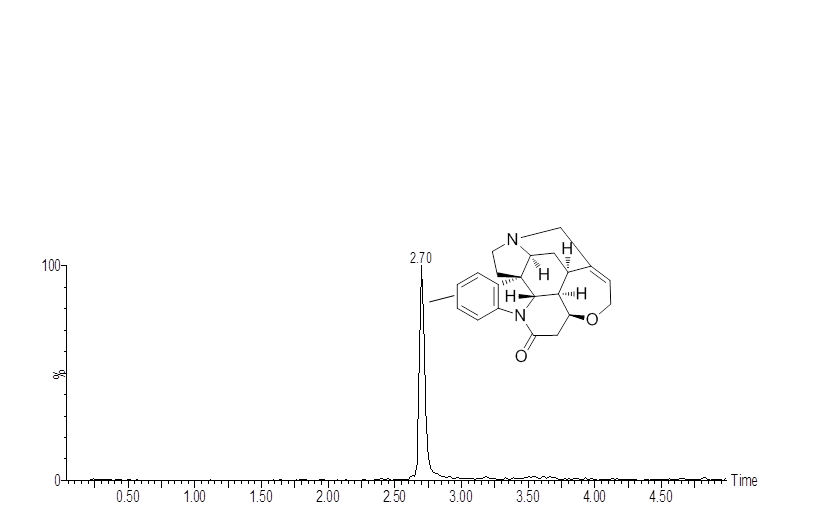
**

**Dihydroxystrychnine**

**Pseudostrychnine**

**Pseudobrucine**

**Brucine**

**Strychnine**


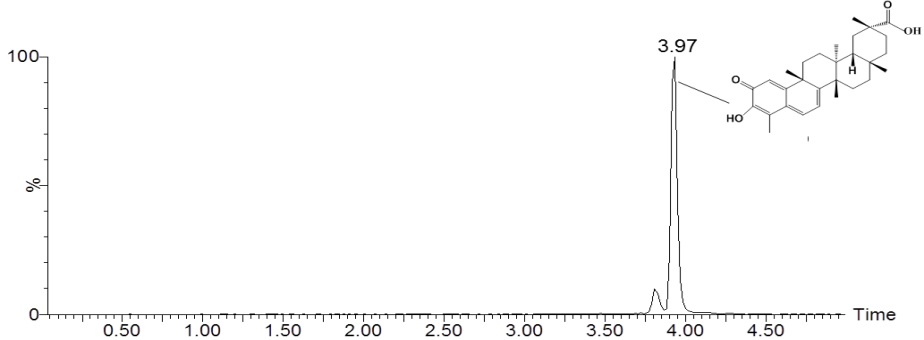

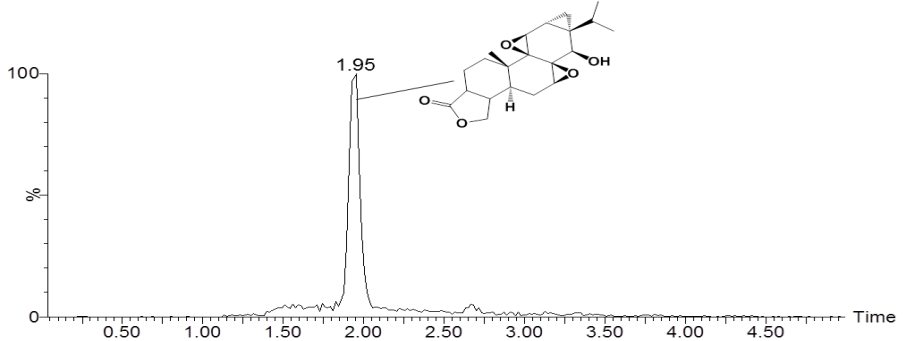
**Figure S2: Representative chromatograms of triptolide and celastrol in TGT** **suspension derived from UPLC-QTOF/MS analysis. The insert shows chemical struct**

**Celastrol**

**Triptolide**
